# Kinetics of CD4^+^ T Helper and CD8^+^ Effector T Cell Responses in Acute Dengue Patients

**DOI:** 10.3389/fimmu.2020.01980

**Published:** 2020-09-24

**Authors:** Dao Huy Manh, Lan Nguyen Weiss, Nguyen Van Thuong, Shusaku Mizukami, Shyam Prakash Dumre, Quang Chan Luong, Le Chi Thanh, Cao Minh Thang, Pham Thanh Huu, Le Hong Phuc, Cao Thi Hong Nhung, Nguyen Thi Mai, Nguyen Quang Truong, Vu Thien Thu Ngu, Do Kien Quoc, Tran Thi Ngoc Ha, Tran Ton, Tran Van An, Oday Halhouli, Le Nhat Quynh, Mohamed Gomaa Kamel, Juntra Karbwang, Vu Thi Que Huong, Nguyen Tien Huy, Kenji Hirayama

**Affiliations:** ^1^Department of Immunogenetics, Institute of Tropical Medicine (NEKKEN), Nagasaki University, Nagasaki, Japan; ^2^Graduate School of Biomedical Sciences, Nagasaki University, Nagasaki, Japan; ^3^Department of Immunology and Microbiology, Pasteur Institute, Ho Chi Minh City, Vietnam; ^4^Department of Clinical Product Development, Institute of Tropical Medicine (NEKKEN), Nagasaki University, Nagasaki, Japan; ^5^National Program for Dengue Control, Pasteur Institute, Ho Chi Minh City, Vietnam; ^6^HIV Laboratory, Pasteur Institute, Ho Chi Minh City, Vietnam; ^7^Nguyen Dinh Chieu Hospital, Ben Tre, Vietnam; ^8^Faculty of Medicine, The University of Jordan, Amman, Jordan; ^9^Online Research Club (www.onlineresearchclub.org/), Nagasaki, Japan; ^10^Hue University of Medicine and Pharmacy, Hue, Vietnam; ^11^Faculty of Medicine, Minia University, Minya, Egypt

**Keywords:** dengue, T helper cells (Th cells), cytokine, T cell subsets, cytoxic T cells

## Abstract

**Background:** The protective or pathogenic role of T lymphocytes during the acute phase of dengue virus (DENV) infection has not been fully understood despite its importance in immunity and vaccine development.

**Objectives:** This study aimed to clarify the kinetics of T lymphocyte subsets during the clinical course of acute dengue patients.

**Study design:** In this hospital-based cohort study, 59 eligible Vietnamese dengue patients were recruited and admitted. They were investigated and monitored for T cell subsets and a panel of clinical and laboratory parameters every day until discharged and at post-discharge from the hospital.

**Results:** We described for the first time the kinetics of T cell response during the clinical course of DENV infection. Severe cases showed significantly lower levels of effector CD8^+^ T cells compared to mild cases at day −1 (*p* = 0.017) and day 0 (*p* = 0.033) of defervescence. After defervescence, these cell counts in severe cases increased rapidly to equalize with the levels of mild cases. Our results also showed a decline in total CD4^+^ T, Th1, Th1/17 cells during febrile phase of dengue patients compared to normal controls or convalescent phase. On the other hand, Th2 cells increased during DENV infection until convalescent phase. Cytokines such as interferon-γ, IL-12p70, IL-5, IL-23, IL-17A showed tendency to decrease on day 0 and 1 compared with convalescence and only IL-5 showed significance indicating the production during acute phase was not systemic.

**Conclusion:** With a rigorous study design, we uncovered the kinetics of T cells in natural DENV infection. Decreased number of effector CD8^+^ T cells in the early phase of infection and subsequent increment after defervescence day probably associated with the T cell migration in DENV infection.

## Background

Dengue has emerged as a major mosquito-borne disease affecting over 100 countries that results in 96 million symptomatic cases, including 500,000 severe cases and 22,000 deaths annually (1–3). Human infection with any serotype of DENV (DENV-1 to –4) produces a broad range of clinical manifestations from mild fever to severe dengue involving hypovolemic shock characterized by plasma leakage (increased vascular permeability), multi-organ failure, and severe bleeding (1, 3). Unfortunately, no specific treatment for dengue is available. The recently licensed dengue vaccine based on humoral immunity induced high levels of neutralizing antibody against four DENV serotypes but showed lower efficacy for DENV-2 (4), which also suggests the potential protective function of T cells during DENV infection (5).

One of the interesting clinical features of DENV infection is that only a small proportion of the dengue patients progress to severe forms following defervescence, while the rest gradually recover (6). This implies the existence of different immune responses upon DENV infection characterized by their outcomes as protection or pathogenesis (7, 8). Several hypotheses including antibody-dependent enhancement (ADE) (7, 9) and T cell mediated immune response have been put forward, although the latter remained more controversial (7, 8). Secondary infection with heterologous DENV serotype induces cross-reactive T cells that lack killing capacity but release pro-inflammatory cytokines making the condition more severe (8, 10). Moreover, cellular immunity (protective and pathogenic) in an acute phase of other viral infections has been well-characterized utilizing the flow cytometry-based analyses (11–13). Thus, there is a need of further understanding in potential dual roles of T cells during DENV infection.

Several studies on animal models have reported the protective roles of T cells (both CD4^+^ and CD8^+^) during DENV infection (14–16). While these observations in animal models are certainly crucial in suggesting the roles of T cell in DENV infection or developing hypotheses, these findings are difficult to extrapolate in actual human infection, which is a major limitation of animal studies. Therefore, a rigorous study design is necessary to uncover the T cell phenomenon in natural human DENV infection, which often requires tremendous efforts. As a consequence, limited information known about T cell roles in human DENV infection except for a few reports that suggested cytotoxic CD4^+^ T cells contribute in controlling DENV infection (17) while others suggested the roles of both CD8^+^ cytotoxic and CD4^+^ helper T cells in DENV infection (7–9, 18). Detailed knowledge of the kinetics of T cell subsets in dengue patients will contribute to improve clinical management and vaccine strategy.

## Objectives

Considering the knowledge gap in differential T cell response during natural DENV infection in human, we carried out a hospital-based prospective study to observe the precise kinetics of T cells during the entire course of illness in mild and severe cases of dengue.

## Study Design

### Ethical Statement

This study was approved by Institutional Review Boards of the Pasteur Institute in Ho Chi Minh City (PIHCM) (No. 602/QD-Pas 27/12/10) and Institute of Tropical Medicine (NEKKEN), Nagasaki University (No. 11063072). The study was conducted according to the Declaration of Helsinki. Written informed consent was obtained from all participants and/or their parent/legal guardian (in the case of minors).

### Study Design and Patient Recruitment

This was a hospital-based prospective study conducted at Nguyen Dinh Chieu Hospital, Ben Tre province, Vietnam, from July 2011 to May 2013. The inclusion and exclusion criteria have been described elsewhere in our previous report (19) and provided in the Supplementary Materials. In our design, after the identification of dengue patients who met the inclusion criteria, they were required to be hospitalized despite having no severe manifestations at the time of recruitment. This was done to ensure stringent monitoring and to eliminate potential biases which could otherwise occur. All the patients were rigorously investigated and monitored by an experienced physician for a panel of clinical and laboratory parameters every day until discharged from the hospital (Supplementary Figure 1). All patients were treated according to the guidelines of the Ministry of Health, Vietnam.

### Dengue Diagnosis

Dengue was screened by DENV NS1 antigen ELISA kit (Bio-Rad Laboratories, Inc., Marnes-la-Coquette, France) and confirmed by either DENV RNA RT-PCR or MAC-ELISA as described elsewhere (19, 20). In-house IgM/IgG capture ELISA assays (PIHCM, Vietnam) in paired sera (acute and convalescent) were performed to identify primary and secondary dengue infections where the secondary was defined as IgM/IgG ratio <1.8 and ≥4-fold rise of the IgG, respectively (20, 21).

### Blood Sampling Strategy for Kinetic Profiles

Blood samples were collected each day from the enrolled patients until discharge. One additional sample was also taken two weeks after discharge (post-discharge) from each patient (Supplementary Figure 1). Whole blood was used for T cell subset analysis and routine hematological parameters, while the plasma (multiple aliquots) was used for other laboratory profiles. All samples were transferred to PIHCM within 24 h after collection.

### Healthy Controls

Control samples were taken from nine healthy Vietnamese donors, from a highly endemic area and the same Kinh ethnic group, without current or recent history of fever or any other disease symptoms. To rule out DENV infection, DENV NS1 antigen and DENV-specific IgM antibody in the plasma were tested by ELISA as described above.

### Patient Monitoring and Clinical Outcome

Each patient was monitored daily as in-patient and all the clinical data were duly recorded which include, but are not limited to, blood collection time, clinical manifestations [vomiting, hemorrhagic tendencies (such as mucosal, gastrointestinal, menstruation, nosebleed etc.), liver enlargement, and progression to severe syndromes, e.g., shock], treatment history, and laboratory parameters (hematocrit level, platelet count, leukocyte count, etc.). Clinical outcome was then linked back to the patient data. Clinical outcome was classified based on the established criteria as described previously (22–29) and compatible with the WHO 2009 classification for severity grading (1). For T cell kinetic analysis, the day of defervescence was designated as day 0, which was defined as the first time when a patient's body temperature dropped below 38°C for a 24 h period. One day before defervescence was assigned as day −1, while 1 day after defervescence was assigned as day 1 and so on. Post-discharge indicated 15–33 days after defervescence (Supplementary Figure 1).

### Flow Cytometry

The absolute number of CD4^+^ and CD8^+^ T cells were measured using TBNK Multi 6-colors BD kit (BD, San Diego, CA) according to the manufacturer's protocol (Supplementary Figure 2). To identify T cell subsets, these surface markers were analyzed using the following antibodies: CD3-APC/Cy7 (SK7), CD4-PerCP/Cy5.5 (RPA-T4), CD8-PerCP/Cy5.5 (RPA-T8), CD45RA-FITC (5H9), CD45RO-FITC (UCHL1), CXCR3-APC (1C6), CCR6-PE (11A9), CCR7-PE (150503), and CD62L-APC (DREG-56) (BD). Briefly, whole blood was stained with these fluorochrome-conjugated antibodies for 30 min and treated with lysis solution (BD) for 20 min at room temperature. After rinsing with phosphate buffer (pH 7.2–7.4), cells were acquired by FACS Canto II (BD). The gating strategy for flow cytometric analysis has been given in Supplementary Figures 2, 3. Analysis of flow cytometry data was accomplished with FlowJo version 10.0 (FLOWJO, LCC, Ashland, OR). Based on the cell surface markers, percentage of Th1 (CD3^+^, CD4^+^, CD45RA^−^, CXCR3^+^CCR6^−^), Th2 (CD3^+^, CD4^+^, CD45RA^−^, CXCR3^−^CCR6^−^), Th1/17 (CD3^+^, CD4^+^, CD45RA^−^, CXCR3^+^CCR6^+^), Th17 (CD3^+^, CD4^+^, CD45RA^−^, CXCR3^−^CCR6^+^) (17, 30, 31), and effector CD8^+^ T cell (CD3^+^, CD8^+^, CD45RO^−^, CCR7^−^CD62L^−^) (32– 34) were calculated (Supplementary Figures 2, 3).

The absolute cell number in each T cell subset was calculated by multiplying the percentage of each subset in activated CD4^+^ or effector CD8^+^ with the absolute number of CD4^+^ and CD8^+^ cell, respectively, which were counted with TBNK Multi 6-colors BD kit.

### Plasma Cytokines

For the quantification of plasma cytokines, MILLIPLEX MAP kits (Human Cytokine/Chemokine Magnetic Beads Panel and Human Th17 Magnetic Beads Panel, Millipore) were used. Frozen plasma samples were measured as indicated in instructions of the kits. Data were analyzed with LABScan 100 (Luminex).

### Statistical Analyses

Data were analyzed with R version 3.3.2. The two groups were compared using Fisher's exact or Chi-square test for categorical variables, and Wilcoxon rank sum test for continuous variable (T cell data). Significant difference was determined when *p* < 0.05. Graphs were presented as line and LOESS curve fitting (35). Post-discharge data were presented as dot blot with the median bar while control data were presented as solid triangle with median bar.

## Results

### Demographic, Clinical, and Laboratory Features of Dengue Patients

**Figure 1:**
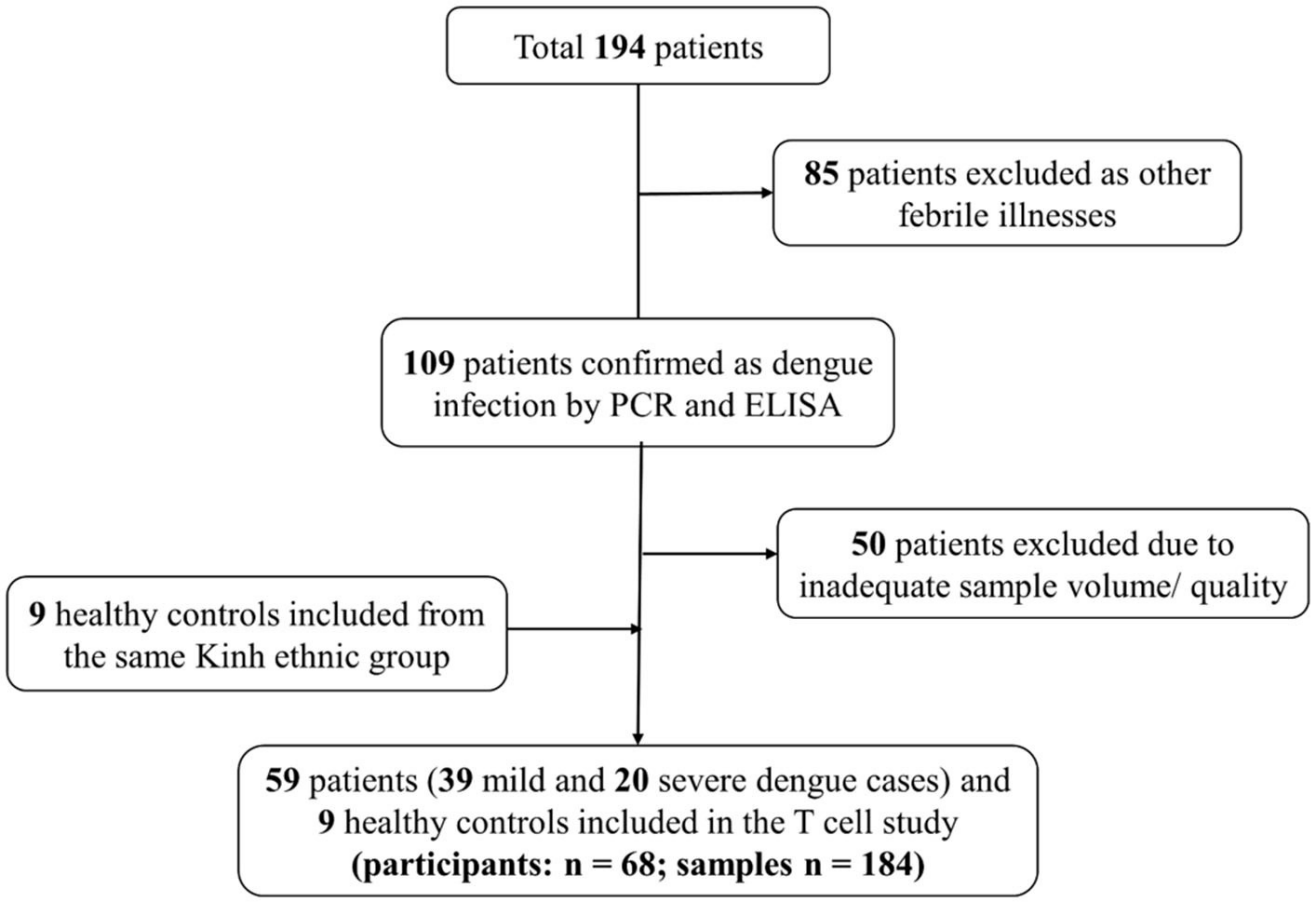
Flow diagram illustrating patient selection and sample investigation. Fifty-nine symptomatic dengue patients were enrolled. Blood samples were collected each day from the enrolment until discharge. One sample was also obtained 2 weeks after discharge as convalescent sample. T cell subsets were analyzed using these samples (*n* = 184).

To understand the kinetics of T cell in DENV infection, a total of 175 samples (115 samples from mild and 60 from severe patients) collected every day from day −4 before defervescence to post-discharge day were analyzed by flow cytometry. Additionally, nine healthy controls samples from the epidemic area were also examined (Figure 1, Supplementary Table 1). There were no significant differences for participant age, gender, and hemorrhagic tendencies between the three groups (mild dengue, severe dengue, and healthy control). The severe dengue group had significantly higher increase in hematocrit levels (*p* < 0.05). Only two patients had liver enlargement (>2 cm) and both were from the severe dengue group. Other clinical features such as primary and secondary infection or thrombocytopenia were not different between mild and severe patients, and so was the distribution of different serotypes. DENV-2 accounted for 40% of severe cases compared to 28.2% in mild cases (non-significant, Table 1). Samples from a total of 59 patients were applied to T cell analysis.

**Table 1:** Demographic and clinical features of T cell study subjects.

**Characteristic**	**Mild**	**Severe**	**HC**	*p*-value*
	***n* = 39**	***n* = 20**	***n* = 9**	
Age—median years (IQR)	14 (12–22.5)	13 (10–17.3)	35 (31–41)	0.2081
Male (%)	15 (38.5)	10 (50)	6 (66.6)	0.419
Primary infection (%)	10 (25.6)	7 (35)		0.5474
Secondary infection (%)	29 (74.3)	13 (65)		0.5474
Percent increase in Htc—median (IQR)	13 (5.7–21.5)	23 (14.2–28.6)		0.0092
Hemorrhagic tendencies (%)	25 (64.1)	15 (75)		0.5577
Persistent vomiting (%)	1 (2.6)	3 (15)		0.1083
Liver enlargement >2 cm (%)	0 (0)	2 (10)		0.111
Thrombocytopenia (%)	15 (38.5)	13 (65)		0.0616
**DENV serotype**				
DENV 1 (%)	6 (15.4)	6 (30)		0.3049
DENV 2 (%)	11 (28.2)	8 (40)		0.3903
DENV 3 (%)	3 (7.7)	2 (10)		1
DENV 4 (%)	14 (35.9)	5 (25)		0.5577

IQR, Interquartile range; Htc, Hematocrit level; DENV, Dengue virus; HC, Healthy control; Thrombocytopenia, Platelet count <100 × 10^9^ cells/L, *Comparison between Mild and Severe group was done using Fisher's exact test (F) or Chi square test for categorical variables and Wilcoxson rank sum test for continuous variables.

### Kinetic Profiles Revealed Lower Effector CD8^+^ T Cells in Severe Dengue Patients Compared to Mild Dengue Patients Before and During the Defervescence

**Figure 2:**
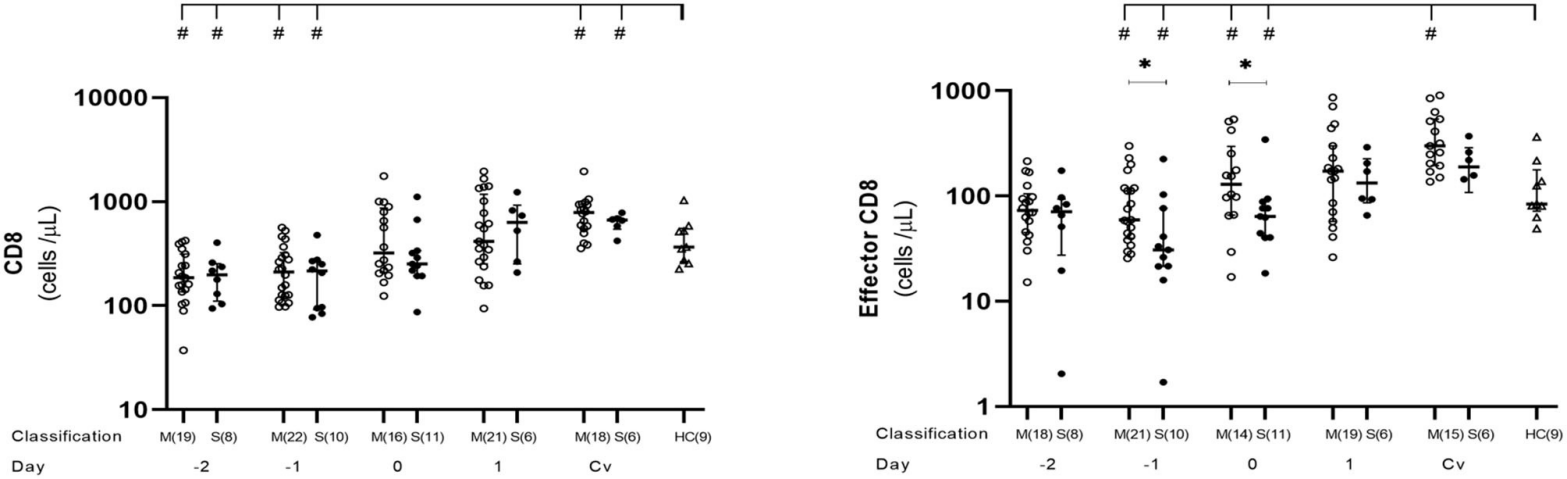
Kinetics of CD8^+^ and effector CD8^+^ T cells in mild and severe dengue infection. Number of CD8^**+**^ and effector CD8^**+**^ T cells from dengue patients was monitored in acute and convalescent phase. Open and closed circles show number of cell count from mild dengue patients and severe dengue patients, respectively. Open triangle shows data from healthy control. Asterisks (*) indicate significant difference (*p* < 0.05) between mild and severe group on a single day. Hash (#) indicates significant difference (*p* < 0.05) between healthy control and other groups. M and S indicate mild and severe dengue patients, respectively. Number in parentheses indicates number of samples in each time point. Day 0 denotes defervescence day. Cv and HC indicate convalescent phase and healthy control, respectively.

We conducted kinetic T cell subset analysis from day −4 to 5 and during the convalescent phase of acute DENV infection. Number of samples examined each day varied from 1 to 22, as shown in Supplementary Table 1 and each figure. CD8^+^ T cells generally contribute to the antiviral response by directly killing infected cells. The kinetic profiles of CD8^+^ and effector CD8^+^ T cells during the course of DENV infection showed diminished level of absolute total CD8^+^ counts compared to healthy controls in both mild and severe cases during the febrile phase, which continued to incline, reaching normal level around day 0 and further increased to remain higher than normal levels until the convalescent phase (Figure 2, Supplementary Table 1). CD8^+^ T cell counts at post-discharge period were significantly higher in both mild (*p* = 0.009) and severe (*p* = 0.0496) groups compared to healthy controls. Effector CD8^+^ cells were significantly lower in severe than mild cases at day −1 (*p* = 0.017) and day 0 (*p* = 0.033). However, after defervescence, effector CD8^+^ T cell counts in severe cases equalized with the levels of mild cases, and high levels of the effector CD8^+^ count remained until convalescence (Figure 2, Supplementary Table 1).

### Lower Level of Th1, Th17, and Th1/17 CD4^+^ T Cells Detected in Dengue Patients Before Defervescence Compared to Healthy Controls

**Figure 3:**
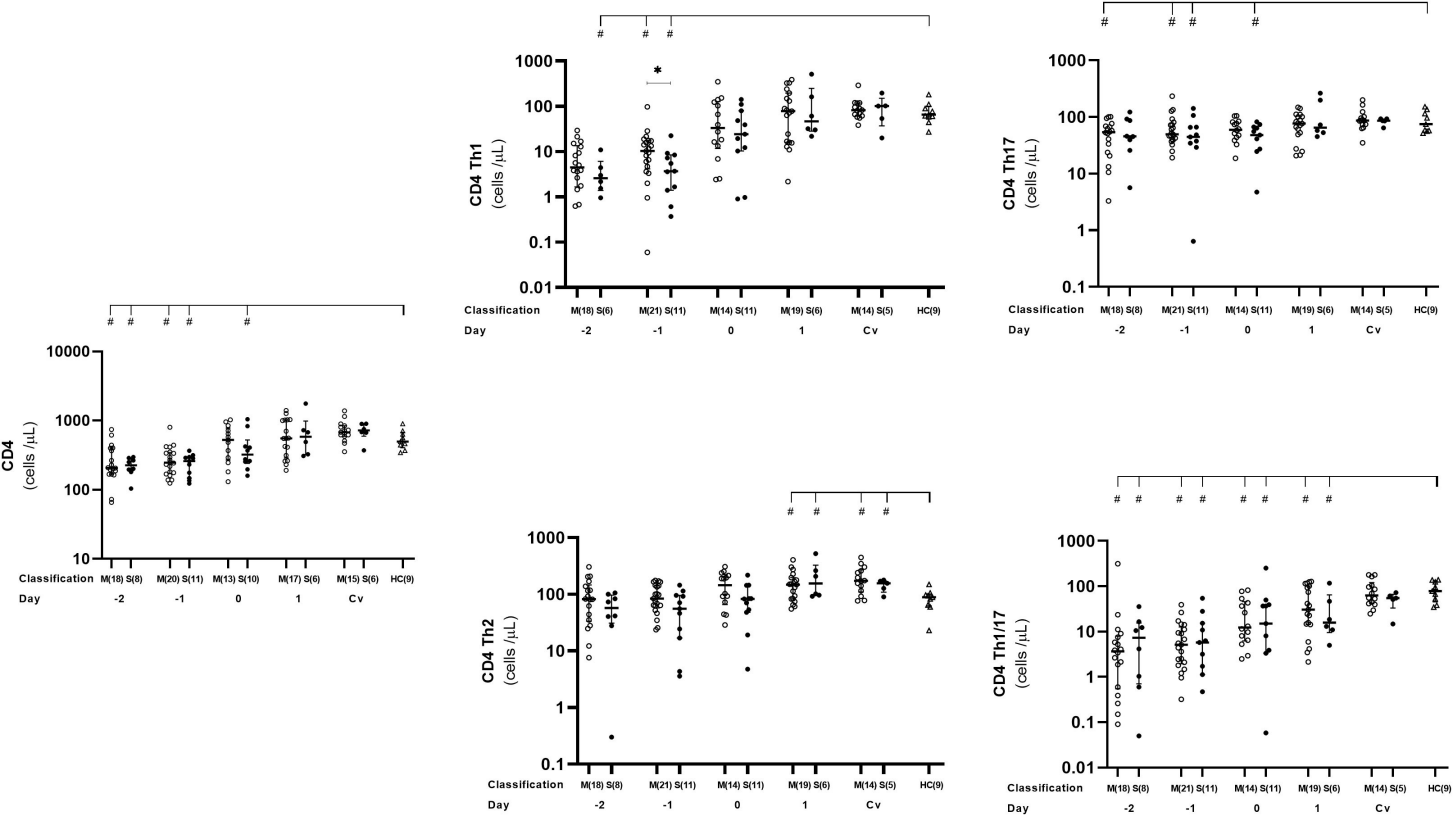
Kinetics of CD4^+^, Th1, Th2, Th1/17, and Th17 T cells in mild and severe dengue infection. Number of CD4^**+**^, Th1, Th2, Th1/17, and Th17 T cells from dengue patients was monitored in acute and convalescent phase. Open and closed circles show number of cell count from mild dengue patients and severe dengue patients, respectively. Open triangle shows data from healthy control. Asterisks (*) indicates significant difference (*p* < 0.05) between mild and severe group on a day. Hash (#) indicates significant difference (*p* < 0.05) between healthy control and other groups. M and S indicate mild and severe dengue patients, respectively. Number in parentheses indicates number of samples in each time point. Day 0 denotes defervescence day. Cv and HC indicate convalescent phase and healthy control, respectively.

We observed here that absolute number of activated CD4^+^ as well as Th1 and Th17 cells behaved in the same trend. Overall, the number of these cells were significantly lower in dengue cases than that in the healthy controls before day 0 (Figure 3, Supplementary Table 1), which then reached normal levels at day 1 and remained stable until convalescent phase. At day −1, severe cases showed significantly lower activated Th1 counts than in the mild cases (*p* = 0.0498) (Figure 3, Supplementary Table 1).

We also analyzed Th1/17 (CCR6+CXCR3+) cells which were originally related to autoimmunity, but recently turned out to be significant in microorganism infections including dengue virus (17, 35, 36). We noticed that kinetics of Th1/17 cells was comparable to Th1 and Th17, with lower counts in dengue patients compared to that in healthy controls during the early phase of infection from day −2 until 1 (severe cases) or day −4 (mild cases). Interestingly, mild patients had significantly lower number of Th1/17 cells than severe ones on day 3 (*p* = 0.03; Figure 3, Supplementary Table 1).

While overall CD4^+^ T cell subsets were lower at the early phase of dengue infection, then increased toward normal level after defeverescence and maintained until convalescence, Th2 cells behaved differently. Dengue infection cases had significantly higher levels of Th2 cells than normal at day 1 and remained high until convalesce (*p* = 0.036 and 0.019, respectively).

### Plasma Cytokine Dynamics During Acute Dengue Infection

**Figure 4:**
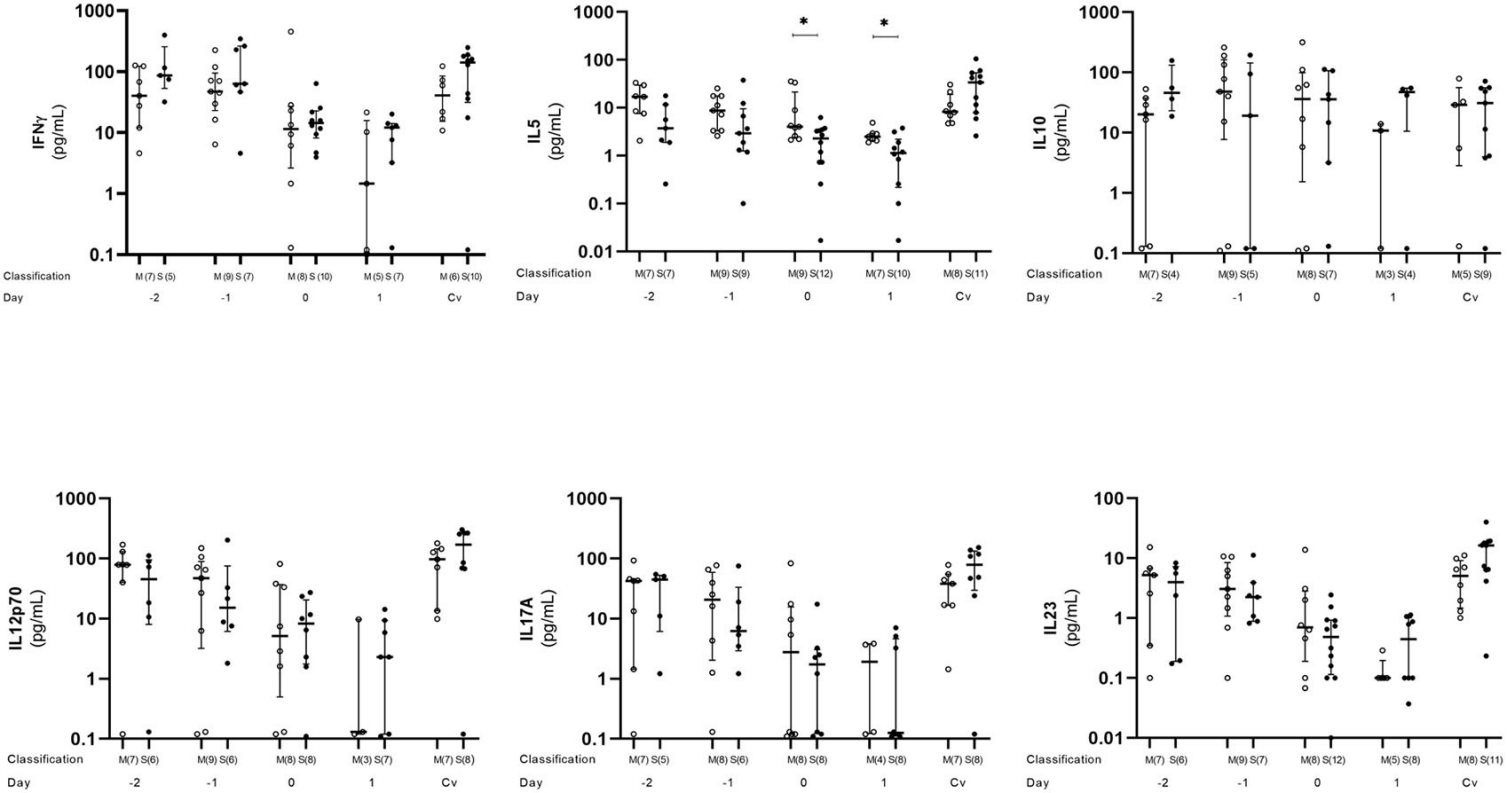
Kinetics of plasma cytokine in dengue infection. Cytokines from dengue patients' plasma were monitored in acute and convalescent phase. Open and closed circles show number of cell count from mild dengue patients and severe dengue patients, respectively. Open triangle shows data from healthy control. Asterisks (*) indicates significant difference (*p* < 0.05) between mild and severe group on a single day. M and S indicate mild and severe dengue patients, respectively. Number in parentheses indicates number of samples in each time point. Day 0 denotes defervescence day. Cv and HC indicate convalescent phase and healthy control, respectively.

Plasma level of five cytokines (IFN-γ, IL-5, IL-10, IL-12p70, IL-17A, and IL-23) were estimated. All cytokines except IL-10 showed a similar tendency with the plasma level decreasing from day −2 to 1, with day 1 as the lowest level. After that, the plasma level started to increase and became similar to or higher than day −2 at convalescent phase. On the other hand, IL-10 did not show such a trend, and the plasma levels were almost similar on every time point. When compared between mild and severe cases, IL-5 plasma levels was statistically significant difference (*p* < 0.05) at day 0 and 1 (Figure 4).

## Discussion

Significant effort has been invested over the last two decades in order to understand the input of DENV-specific T cells in protection or enhanced immunopathology. In general, CD8^+^ T cells can eliminate viral infection by generating effector cells, induce cytotoxicity, IFN-γ, and TNFα (37). CD4^+^ T cells indirectly control infection by enhancing B and CD8^+^ T cell activation, memory to control recurring pathogen, and production of inflammatory and anti-viral cytokines (38).

HLA restricted CD4^+^ and CD8^+^ T cells are activated upon viral infection and several epitopes have been identified (39). Multiple lines of evidence suggest that CD4^+^ and specially CD8^+^ T cells importantly contributed against DENV infection in mouse models (15, 16) and in humans (39–41). In many recent human studies (39–41), analysis was conducted on blood collected from a healthy donor in the endemic area. Here, we characterized for the first time the precise kinetics of T cell subsets in the meticulously monitored cohorts of dengue patients.

In this cohort, CD8^+^ and CD4^+^ T cells showed similar kinetic patterns. All DENV infection cases had a tendency of lower number of total CD8^+^, effector CD8^+^, Th1, and Th1/17 cells than those in normal control before the defervescence day which then started to increase till post-discharge period and, interestingly, the increase seems to be faster in severe than mild patients. This emphasized the protective role of the CD8^+^ and CD4^+^ T cells in natural human dengue infection. After activation, CD8^+^ cells possibly migrate to sites of infection and kill infected cells. While CD4^+^ cells provide help to innate immune responses, B cells, and CD8^+^ cells contribute to eliminate DENV from infected tissues (42).

Multiple studies discovered that not only CD8^+^ but also CD4^+^ T cells appeared to contribute in protecting DENV infection through cytotoxic functions such as antigen-specific killing targeting non-structural proteins (43). Additionally, CD4^+^ T cells interfere the lysis of non-antigen-presenting bystander target cells (16) via two main pathways: perforin-dependent and FAS/FAS ligand (44). Cytotoxic activity and protecting role of DENV specific CD4^+^ T cells could strengthen the finding from our laboratory and others the association of HLA class II molecules DRB1^*^09:01 (Vietnamese) and DRB1^*^04:01 (Sri Lankan) (45, 46). Th1/17 clone helped antibody production by B cells and to display cytotoxic activity (47). In our patients, a remarkable significant movement of Th1/17 cells during acute DENV infection was observed highlighting their protective role. This finding opens an important area of research as previous studies reported. Th1/17 cells, together with Th1 and other Th1-like cells, showed to be significantly activated (17, 36). As they are the INF-γ producers, their activation could lead to cytokine storm and severity (36).

Contrary to Th1, Th17, and Th1/17 cells, Th2 cells increased from defervescence day until convalescence and were significantly higher than normal levels in both mild and severe cases (Figure 3). A study by Chng et al. (36) had detected this difference in Th2 frequency which was an upward trend during the recovery phase. The increased level of Th2 cells during defervescence period in dengue cases is correlated with our previous finding of elevated levels of plasma mast cell granule-derived factors. Increased vascular permeability, the hallmark of severe dengue, is thought to be provoked by mast cells (48). We and others have previously shown the important role of mast cell-derived mediators, including vascular endothelial growth factor (VEGF), tryptase, chymase, in vascular permeability in dengue severity (19, 48, 49). However, it is still unknown why the onset of vascular leakage occurs during defervescence.

Higher levels of inflammatory cytokines including IFN-γ in acute phase than in defervescence phase were reported (36, 50). Because our study focuses on T cells and the subsets, inflammatory cytokines (e.g., IL-1β, TNF-α) were not well-covered in analysis. We revealed a similar tendency on a major CD8^+^ T cell cytokine IFN-γ, and cytokines represent several helper subsets (Th1, Th2, and Th17) (Figure 4). Decrease of plasma level between day −2 and 1 as a common trend for cytokines except IL-10 and no significant cytokine surge was observed. Our observation strongly suggested that the T cell mediated immunological reaction provoked by dengue virus infection is highly limited in the lesion and is difficult to see from systemic circulating blood levels. To have a clear conclusion, further study will be required.

Looking into the differences between clinical groups, we observed significant lower levels of effector CD8^+^ (at days −1 and 0) (Figure 2), as well as CD4^+^ Th1 (at days −1) in severe group than in mild group (Figure 3). CD4^+^ Th2 cells also behaved the same way between the two groups even though it was not statistically significant (Figure 3). Clinically, the critical phase of dengue begins at day of defervescence (6–9). It is reasonable to speculate that around day-1 is critical time of kinetic T cells which in turns could possibly control the clinical outcome of the infection.

In both groups, effector CD8^+^ increased after day 0, probably due to the migration of these cells to infected tissues and organs before day 0, as seen with the Th1 population. In a study on T cell epitope reactivity against DENV proteome, specific CD4^+^ and CD8^+^ T cells recognized distinct viral proteins confirming the crucial roles of CD4^+^ Th1 in INF-γ production (18). CD4^+^ Th1 subset was claimed to have a positive interaction with naïve CD8^+^ to become effectors (51, 52).

The combination of total CD8^+^, effector CD8^+^, Th1, Th1/17 subsets during the early stage is important to inhibit viral replication in the host cells of tissue sites. The later increment of these cells in the peripheral circulation after defervescence might indicate their return from the infected tissues. There are reports of T cell homing to the skin during the early phase of DENV (53) and HSV-2 infections (54, 55). Our observation of early phase reduction and delayed regain of these cell levels may also be explained by the same phenomenon of committed T cells homing markers to the original skin tissue or inflammation places such as liver, although there is a report that CD8^+^ T cell activation does not enhance prior to the commencement of resolution of viremia or hemo-concentration (39, 53, 56–61).

Secondary heterotypic dengue infection has been associated with severity due to the ineffective viral control of preexisting cross-reactive and low-affinity memory T cells (62). However, the risk of severe dengue in secondary heterotypic infection was not high in our studies (45, 49) including the current study and some others (62). We have also analyzed T cell kinetics in the context of primary and secondary infection (Supplementary Figures 4A, B). At convalescent phase across all studied groups, there was a trend of higher levels of dengue specific or memory T cells in dengue patients compared with healthy controls (Supplementary Figure 4B). The significantly higher activated CD4^+^ and Th17 cells in secondary infection compared with primary infection was revealed at convalescent time (Supplement Figure 4B). This difference occurred by second simulation may be due to some activated memory. The same tendency was also observed in Th1 cells. Th2 cells, however, in both primary and secondary infection cases were higher when compared with that in healthy controls from day 1 until convalescence (Supplementary Figure 4B). As both types of infections similarly increase, perhaps a memory independent response may have occurred. More focused study on the T cell epitopes that are critical for the repeated heterologous infection could reveal the difference (39).

Amino acid differences between DENV serotypes can range from 14 to 67% (63). Recognized T-cell epitopes in the case of sequential heterologous serotype infection can interfere with the outcome from being protective to pathogenic response (63). Out of four serotypes (DENV 1–4), DENV-2 had more severe clinical manifestations than other serotypes in South East Asian and South American populations (45). In this study, although not significant, DENV-2 was still predominant in severe cases while DENV-4 was predominant in the mild cases (Table 1). Engagingly, patients with DENV-4 had the highest CD8^+^ and effector CD8^+^ T cells in convalescent time. It is noteworthy that there were the clusters of severe cases with DENV-2 which had the lowest CD8^+^ (84 and 97 at day −1), as well as effector CD8^+^ T cell count (26, 33, 34 at day −1, and 40, 63, 76 at day 0; Supplementary Figure 5A). Additionally, DENV-2 subjects were found to have the significant higher cell counts (*p* < 0.05) in convalescence across Th1, Th2, and Th1/17 populations (Supplementary Figure 5B).

We observed CD4^+^ and CD8^+^ T cell subset kinetics during the course of natural infection in dengue patients; however, we did not distinguish DENV specific T cell populations that could be analyzed by tetramers (36). Recent findings of Chng et al. showed the full activation and proliferation of all immune subsets, except for B cells in the acute phase of dengue infection. Moreover, by using the strategy of 430 multiplexed peptide-HLA staining, the adaption of T cell during dengue infection revealed specifically to DENV antigen against EBV and influenza's epitopes (36). Additionally, DENV has the T-cell cross-reactivity with zika virus, the other species of Flavivirus (56). We believe that the kinetics of T cells showed here have the specification to DENV, at least with other no relative viruses. We consider these limitations to instruct for our comprehensive future studies. Alternatively, comprehensive data from Weiskopf et al. in the general population from Sri Lanka using multiple peptide technology showed CD8^+^ T cells targeted at non-structural protein epitopes, dominantly at NS3 protein. Meanwhile CD4^+^ T cell epitopes identified at capsid, envelop, and non-structural proteins, thus contributing to protective immunity to DENV infection. Moreover, CD4 ^+^ and CD8 ^+^ T cells increased after a single dose of a tetravalent live-attenuated dengue vaccine were directed to the same region of non-structural protein antigens as T cells from a naturally infected human (17). Those comprehensive HLA reactive T epitope analysis will enhance the availability of dominant tetramers that can be applied to our further analysis.

## Conclusion

With a rigorous study design, we have uncovered the kinetics of T cell in natural dengue infection in our cohorts which, we believe, aids in the understanding of the cellular immune response in dengue. Moreover, the significantly lower number of the effector CD8^+^ cells we observed in severe patients during the febrile phase is probably related to the movement of T cell upon DENV infection (as we hypothesized), which warrants further investigations.

## Data Availability Statement

All datasets generated for this study are included in the article/Supplementary Material.

## Ethics Statement

The studies involving human participants were reviewed and approved by Institutional Review Boards of the Pasteur Institute in Ho Chi Minh City (PIHCM) (No. 602/QD-Pas 27/12/10) and Ethical Review Committee of the Institute of Tropical Medicine (NEKKEN), Nagasaki University (No. 11063072). Written informed consent to participate in this study was provided by the participants' legal guardian/next of kin.

## Author Contributions

LW, VH, SM, NH, and KH conceived and designed the experiments. DM, LW, NVT, SM, SD, CT, CN, NM, NQT, VN, TH, and TT performed the experiments. CN, PH, LP, TA, LW, VH, NH, and KH performed clinical management. DM, LW, QL, LT, NVT, SM, VH, OH, LQ, MK, NH, DQ, SM, SD, KH, and JK analyzed and interpreted the data. All authors wrote and approved the final manuscript.

## Conflict of Interest

The authors declare that the research was conducted in the absence of any commercial or financial relationships that could be construed as a potential conflict of interest.

## Supplementary Material

Supplementary Figure 1 Time frame of blood sample collection. Blood samples were collected each day from the enrolment until discharge. One additional sample was also taken two weeks after discharge (convalescent phase) from each patient.

Supplementary Figure 2 Absolute T cell count using TBNK + TruC Kit. TBNK Multi 6- colours BD- Kit was used to stain whole blood. Major cell populations were defined as CD4^+^ (CD3^+^CD4^+^), CD8+ (CD3^+^CD8+), B (CD19^+^), and NK (CD16^+^CD56^+^). The FACSCanto II automatically calculated the absolute number of each cell type from number of beads run together with cells during flow cytometry.

Supplementary Figure 3 Gating strategy to identify CD4+ and CD8+ T cell subsets. Whole blood was stained by fluorochrome-conjugated antibodies. After that the cells were acquired by FACSCanto II followed by the analysis of data using FlowJo v 10.0. T cell subsets were defined (based on cell surface markers) as: CD8 T cell effector subset (CD3^+^, CD8^+^, CD45RO^−^, CCR7^−^CD62L^−^) **(A)**, and CD4 T cell subsets, Th1 (CD3^+^, CD4^+^, CD45RA^−^, CXCR3^+^CCR6^−^), Th2 (CD3^+^, CD4^+^, CD45RA^−^, CXCR3^−^CCR6^−^), Th1/17 (CD3^+^, CD4^+^, CD45RA^−^, CXCR3^+^CCR6^+^), Th17 (CD3^+^, CD4^+^, CD45RA^−^, CXCR3^−^CCR6^+^) **(B)**. Percentage of each subset was used to calculate absolute number of that subset.

Supplementary Figure 4 T cells kinetics in primary and secondary dengue infection. Number of CD8^+^, effector CD8^+^ T cells (A) and CD4^+^ and CD4^+^ T cell subsets (B) from primary and secondary dengue patients was monitored in acute and convalescent phase. Open and closed circles show number of cell count from primary infection (PI) and secondary infection (SI), respectively. Triangle show data from healthy control. Number in parentheses indicates number of samples in each time point. Day 0 denotes defervescence day. Cv and HC indicate convalescent phase and healthy control, respectively. Asterisks (*) indicates significant difference between primary and secondary infection group on a single day. Hash (#) indicates significant difference (*p* < 0.05) between healthy control and other groups (# *p* < 0.05, ## *p* < 0.01, ### *p* < 0.005, #### *p* < 0.0001).

Supplementary Figure 5 T cells kinetics by virus serotype. Number of CD8+, effector CD8+ T cells (A) and CD4^+^ and CD4^+^ T cell subsets (B) from patient infected with dengue virus 1, 2, 3, 4 serotypes was monitored in acute and convalescent phase. Open circle, triangle, diamond and closed circle show number of cell count from dengue virus 1, 2, 3, 4 serotype, respectively. Asterisks (*) indicates significant difference (*p* < 0.05) between dengue serotypes on a single day. Day indicates day from defervescence day. n indicates number of samples each time point. Cv denotes samples from convalescent phase.

Supplementary Table 1 Absolute T cell count in dengue patients and healthy control.
