# Supplementary figures and images for: Kinetics of CD4+ T Helper and CD8+ Effector T Cell Responses in Acute Dengue Patients
Source: Front Immunol. 2020 Sep 24;11:1980. doi: 10.3389/fimmu.2020.01980 (PMC7542683; doi:10.3389/fimmu.2020.01980)

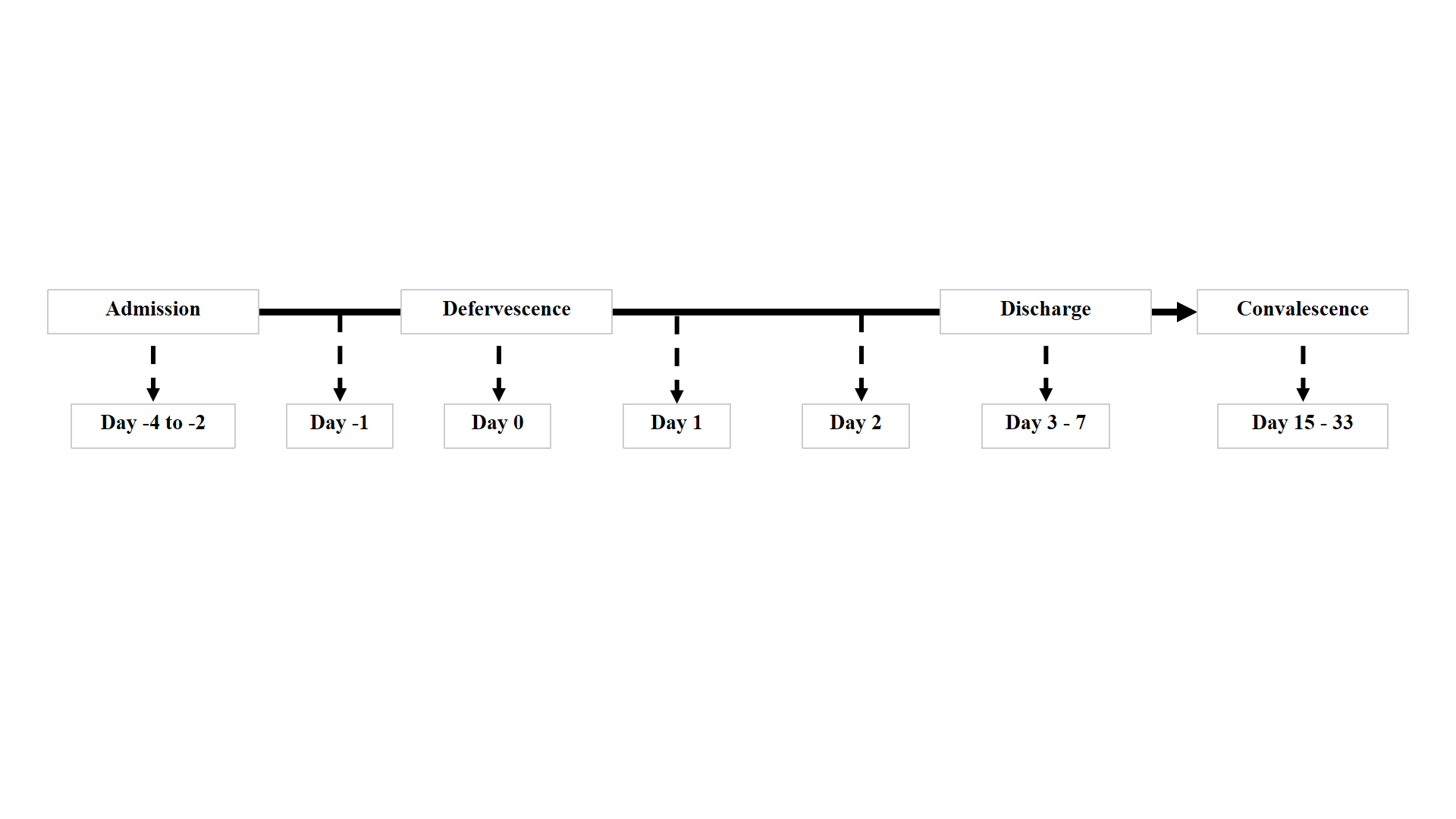

Supplement: Supplementary Figure 1 — Time frame of blood sample collection. Blood samples were collected each day from the enrolment until discharge. One additional sample was also taken two weeks after discharge (convalescent phase) from each patient. [file Data_Sheet_1.zip › Figure S1.tif]

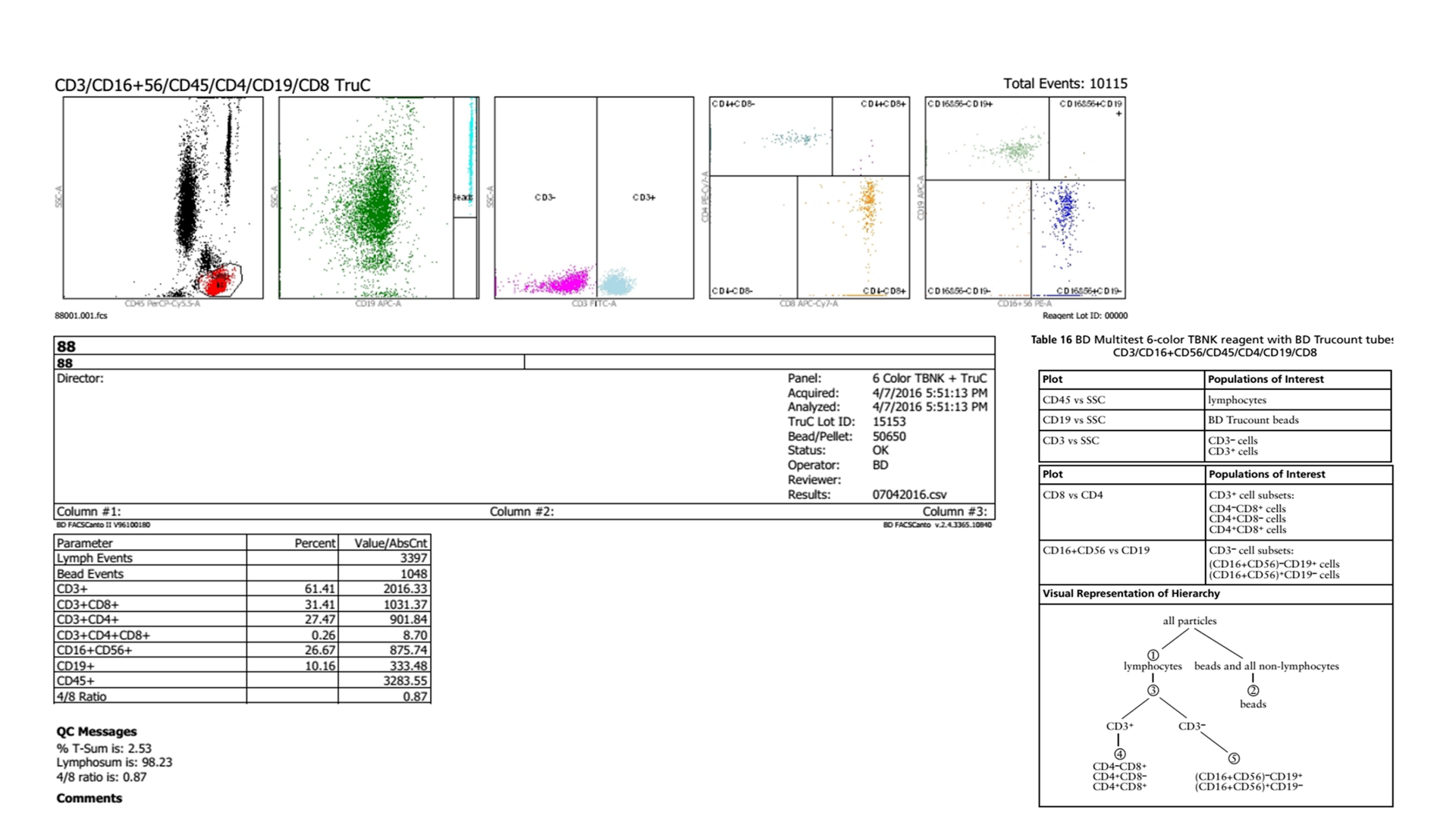

Supplement: Supplementary Figure 1 — Time frame of blood sample collection. Blood samples were collected each day from the enrolment until discharge. One additional sample was also taken two weeks after discharge (convalescent phase) from each patient. [file Data_Sheet_1.zip › Figure S2.tif]

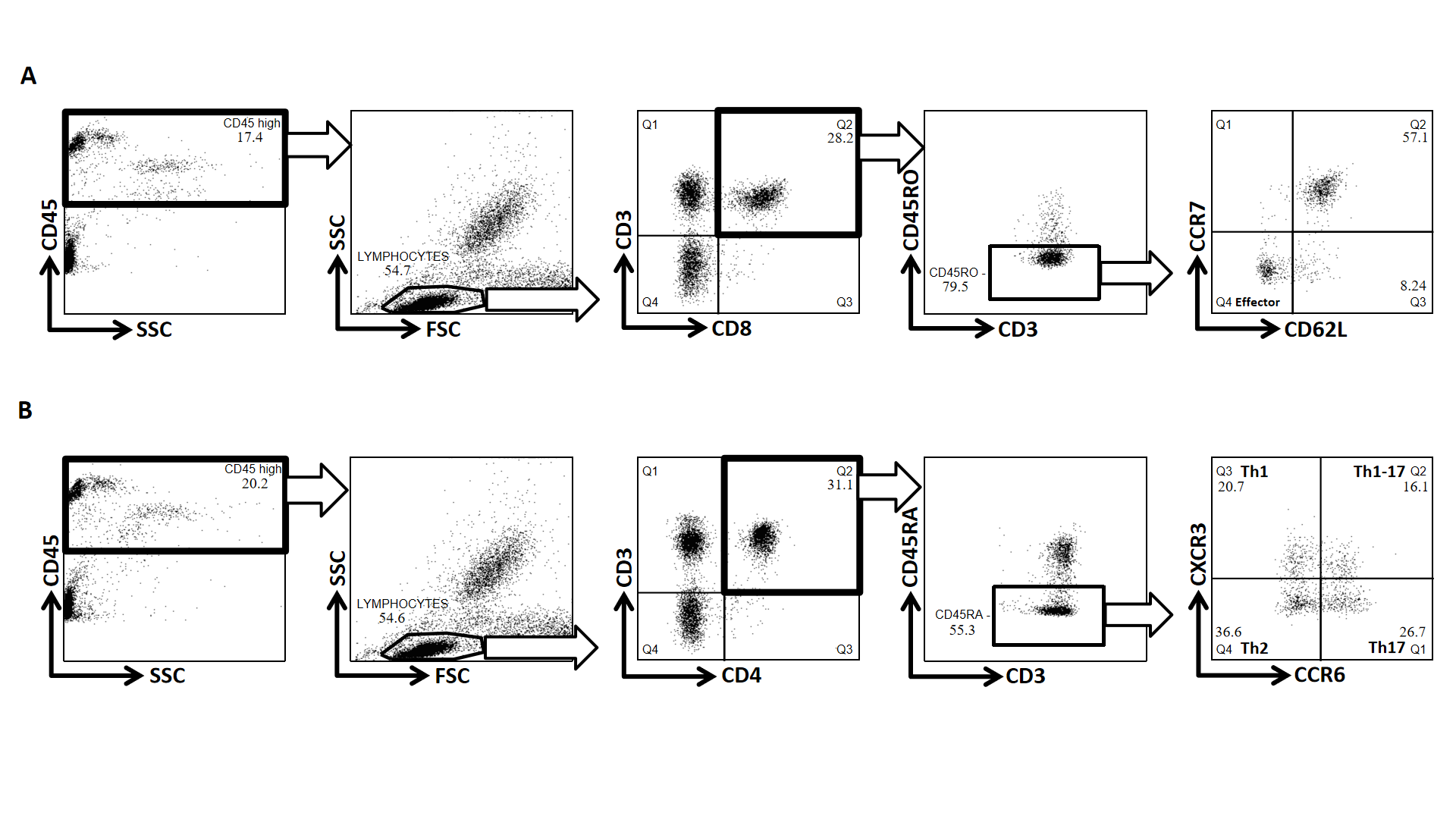

Supplement: Supplementary Figure 1 — Time frame of blood sample collection. Blood samples were collected each day from the enrolment until discharge. One additional sample was also taken two weeks after discharge (convalescent phase) from each patient. [file Data_Sheet_1.zip › Figure S3.tif]

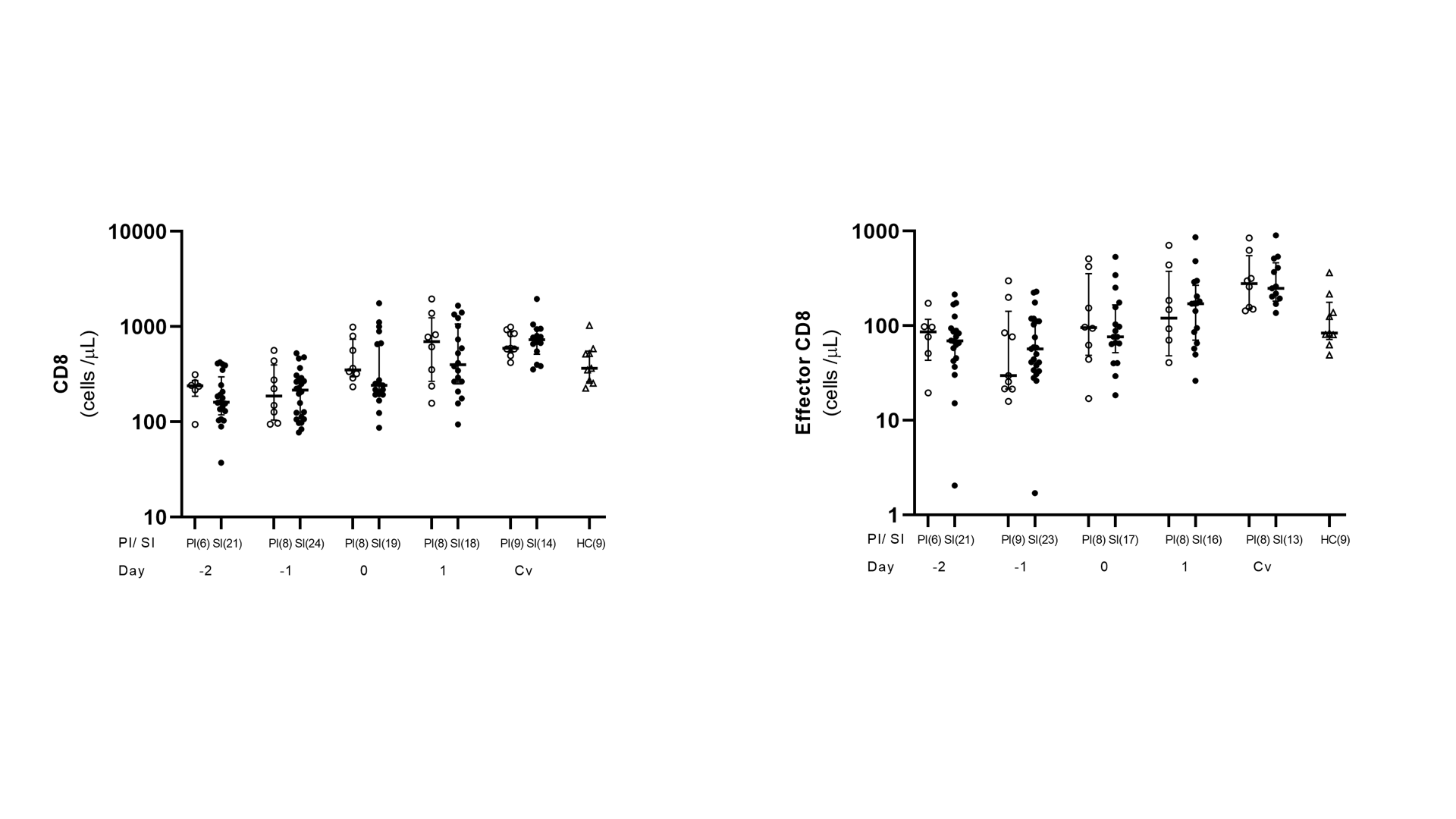

Supplement: Supplementary Figure 1 — Time frame of blood sample collection. Blood samples were collected each day from the enrolment until discharge. One additional sample was also taken two weeks after discharge (convalescent phase) from each patient. [file Data_Sheet_1.zip › Figure S4.tif]

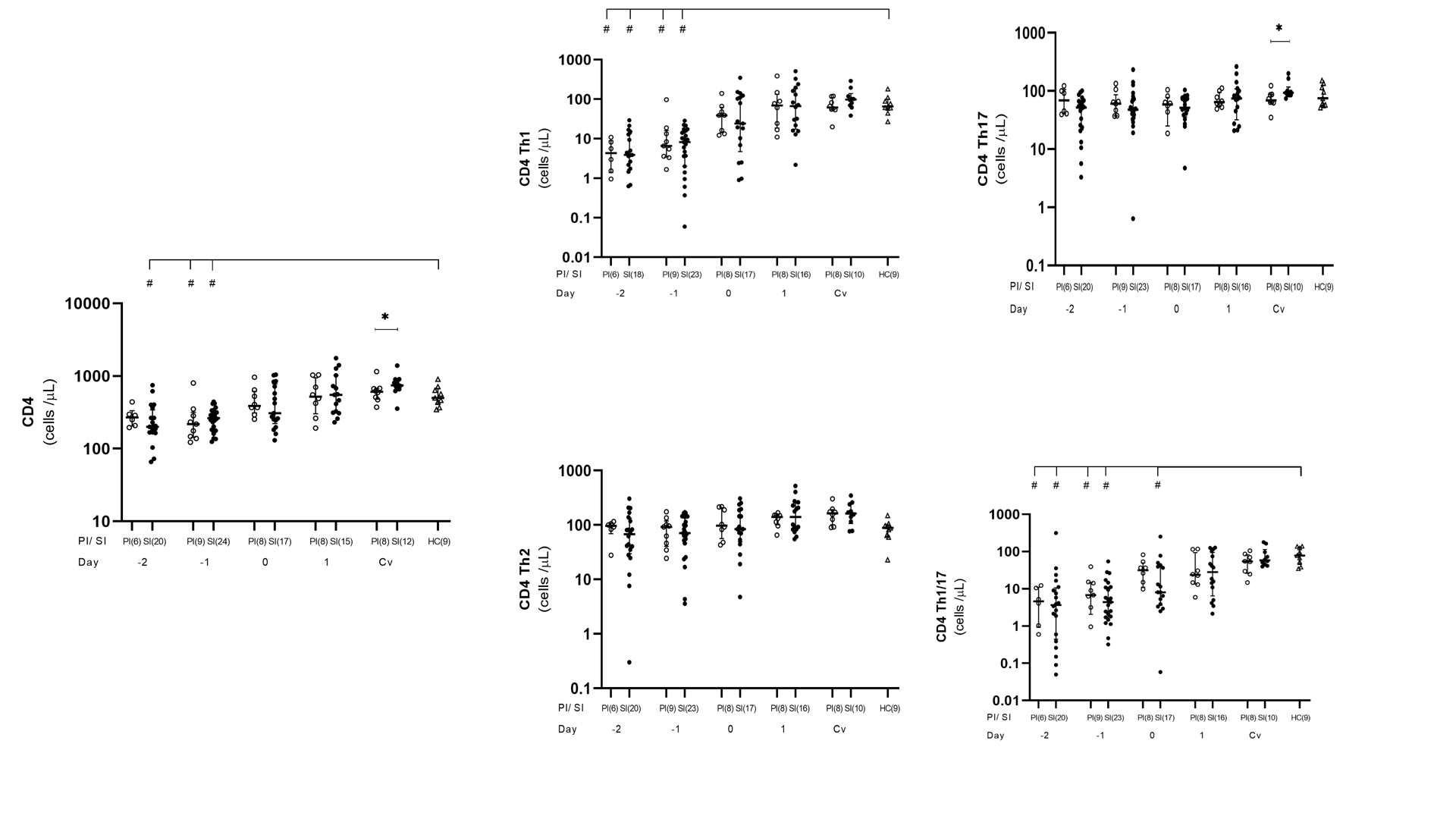

Supplement: Supplementary Figure 1 — Time frame of blood sample collection. Blood samples were collected each day from the enrolment until discharge. One additional sample was also taken two weeks after discharge (convalescent phase) from each patient. [file Data_Sheet_1.zip › Figure S5.tif]

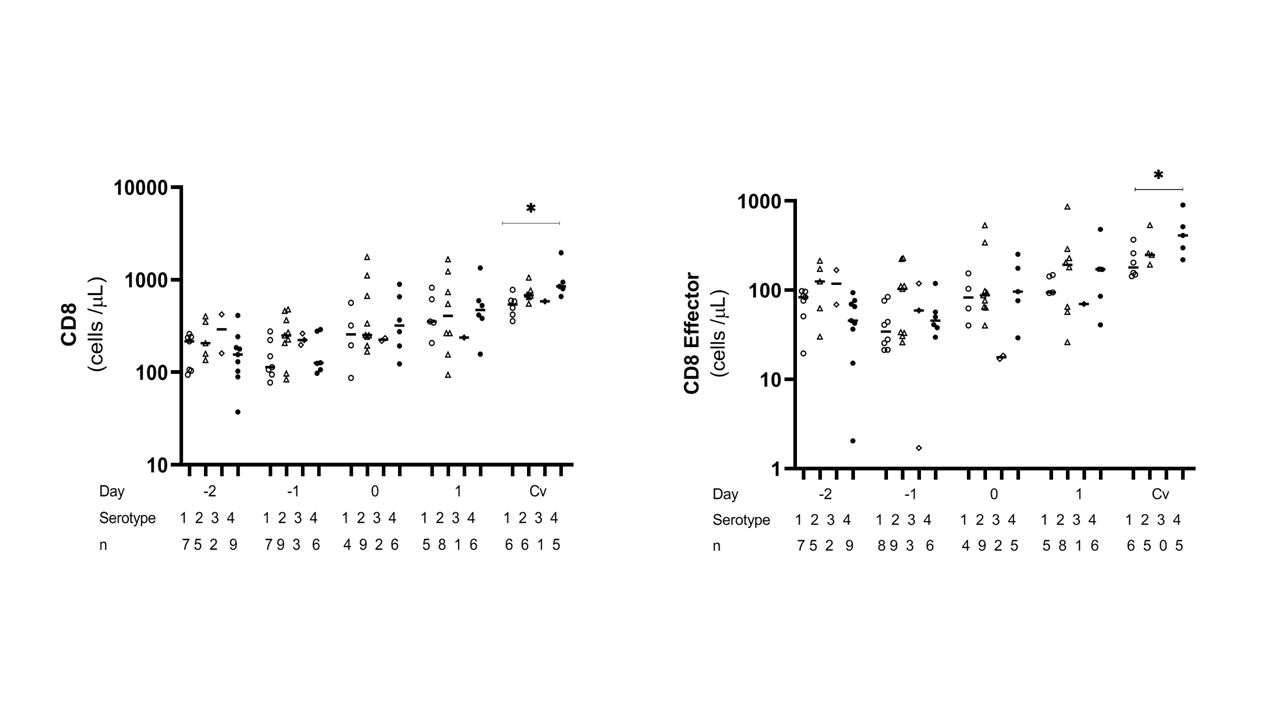

Supplement: Supplementary Figure 1 — Time frame of blood sample collection. Blood samples were collected each day from the enrolment until discharge. One additional sample was also taken two weeks after discharge (convalescent phase) from each patient. [file Data_Sheet_1.zip › Figure S6.tif]

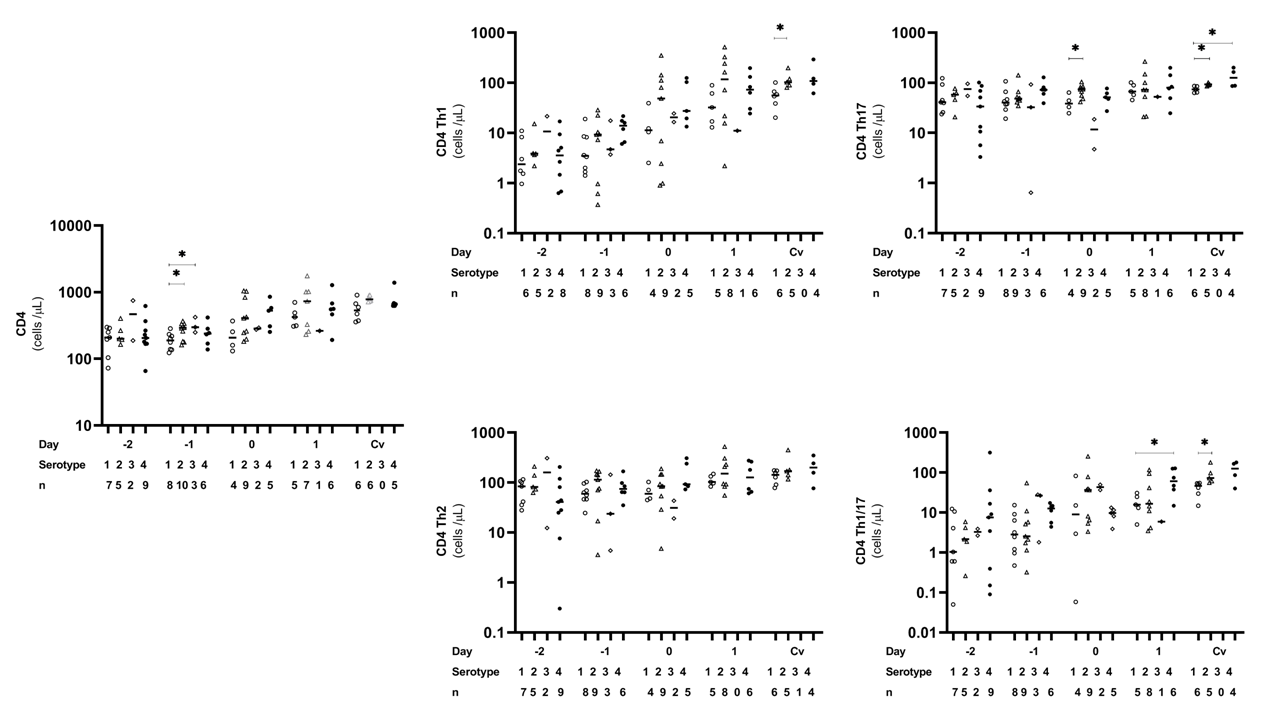

Supplement: Supplementary Figure 1 — Time frame of blood sample collection. Blood samples were collected each day from the enrolment until discharge. One additional sample was also taken two weeks after discharge (convalescent phase) from each patient. [file Data_Sheet_1.zip › Figure S7.tif]
